# How does physicians' educational knowledge-sharing influence patients' engagement? An empirical examination in online health communities

**DOI:** 10.3389/fpubh.2022.1036332

**Published:** 2022-11-07

**Authors:** Xiumei Ma, Pengfei Zhang, Fanbo Meng, Kee-hung Lai

**Affiliations:** ^1^Faculty of Business, Hong Kong Polytechnic University, Kowloon, Hong Kong SAR, China; ^2^School of Political Science and Public Administration, Soochow University, Suzhou, China; ^3^School of Business, Jiangnan University, Wuxi, China

**Keywords:** patients' engagement, physicians' educational knowledge-sharing, registration duration, online health communities, signaling theory

## Abstract

Online health communities (OHCs) are popular channels increasingly used by patients for acquiring professional medical knowledge to manage their own health. In OHCs, physicians provide not only consultation services but also educational medical knowledge to improve patient education. So far, it remains unknown regarding how the educational medical knowledge sharing influence engagement of patients in OHCs. Drawing on the signaling theory, we examined the effects of paid vs. free knowledge-sharing of physicians on patients' engagement behaviors (i.e., patient visit and patient consultation). Data collected from one of the largest OHCs in China show that both paid and free knowledge-sharing are favorable for patients' engagement. Particularly, these two types of knowledge-sharing vary in their impacts. Moreover, physicians' registration duration in OHCs has a positive moderating effect on the relationship between physician's knowledge-sharing and patient engagement. Managers seeking to engage patients at OHCs are advised to share educational medical knowledge to entice them and the patient engagement is more salient for the knowledge shared by physicians active at the platforms for longer time history.

## Introduction

The growth of medical care demand coupled with the shortage of medical resources have become a thorny global issue in many countries. In recent years, the emergence and development of online health communities (OHCs) provide possibility to ease this problem (1). OHCs allow physicians to provide medical services to any patient on electronic platform without time limits and geographic barriers encountered in delivering offline medical services (2). Especially in China, patients in remote areas can access physicians who have registered in OHCs virtually and consult them directly, saving the travel cost and time (3). Meanwhile, patients are enabled to acquire professional medical knowledge from OHCs to better manage their health. Due to these practical advancements, OHCs have become popular service channel to benefit both physicians and patients. For example, registered physicians in OHCs can gain personal benefits and reputations through offering online medical consultation services to patients (4), while patients are enabled to get medical advice, medical information and knowledge, and social support conveniently.

Despite these benefits, OHCs still face some operational challenges for patient engagement. Studies have shown that more than 90% of patients only browse the website without any further engagement behavior (5). Broadly, user engagement behavior can be viewed at two levels, namely shallow engagement and deep engagement (6). In the context of OHCs, patient engagement behavior for medical services includes two steps, visiting physicians' home page (i.e., patient visit) and purchase consultation services (i.e., patient consultation), which correspond to these two levels of engagement. As the consumers of OHCs, visit and consultation behaviors of patients are crucial indicators of success and sustainability of OHCs. Thus, it is critical to understand what factors are desired to facilitate patients' engagement behavior in OHCs.

Most of existing studies on patient engagement in OHCs focused on the effect of patients' psychological perceptions (7, 8). However, OHCs are driven by physicians, thus whether and how physician's activities influence patients' engagement need managerial and research attention. There is also research indicating that information asymmetry between physicians and patients is the key barrier preventing patients from visiting or consulting a physician (3), while physicians' behavior in OHCs can effectively alleviate information asymmetry. In addition to providing consultation services, physicians also share some educational medical knowledge in OHCs. High-quality consultation service obviously attracts more patients, but little is known about how sharing educational medial knowledge influences patient engagement. Physicians' knowledge-sharing in OHCs enables patients to better understand medical knowledge and take proper actions to manage their health, which is extremely valuable for patient education. Based on this significant role, this study aims to explore the impacts of physicians' knowledge-sharing on patients' engagement in OHCs.

Knowledge-sharing has been widely examined in the literature on Q&A platforms and broadly classified into two types: paid knowledge-sharing and free knowledge-sharing (9, 10). In the former case, consumers need to pay for the shared knowledge while everyone can access the shared knowledge freely in case of the latter. In OHCs, physicians' knowledge-sharing refers to the physicians' behavior of publishing educational articles to share medical knowledge to patients, including both paid and free knowledge-sharing. The paid knowledge-sharing behavior of a physician indicates his/her service quality and ability while the free knowledge-sharing behavior represents his/her kindness of offering help. Based on the signaling theory, the knowledge-sharing behaviors can be regarded as signals to diminish the information asymmetry between physicians and patients, thus are supposed to influence patients' choice and behavior decisions (11).

However, most of current research paid more attention on the factors motivating knowledge-sharing behavior but neglected what effects that knowledge-sharing brings, especially in the context of OHCs (12, 13). Moreover, little extant research has empirically examined the effects of these two kinds of knowledge-sharing behaviors within a study. Since paid knowledge-sharing and free knowledge-sharing represent distinct signals, whether they vary in their effects on patients' engagement is unknown. Furthermore, numerous research has emphasized that physicians' characteristics, such as the seniority (14), the ranking (4), and the image (15), play important contingent roles in determining patients' decisions. Nonetheless, the length of physicians' registration duration in OHCs has received little attention. The registration duration demonstrating a physician's level of online service innovation and qualification in OHCs, which may affect patients' evaluation on physicians' behaviors and further influence patients' decisions to engage further with the platform services. Therefore, to fill these research gaps, we address the following specific questions in this study:

Q1: How do both paid and free knowledge-sharing of physicians influence patients' engagement behaviors (e.g., patient visit and patient consultation)?
Q2: What role does physicians' registration duration play in the relationship between physicians' knowledge-sharing and patients' engagement?

To answer these questions, we built up a research model and examined hypotheses by collecting data from the Good Physician Online (www.haodf.com), one of the largest OHCs in China. The results show that both physicians' paid and free knowledge-sharing promote patient visit and patient consultation, but difference exists in the positive effects. In addition, the registration duration has been verified to play significant positive moderating roles. This study provides several contributions to both theory and practice. First, as far as we know, this study is the first to examine patient engagement in OHCs from the perspective of physicians' knowledge-sharing, enriching research on user engagement and literature on OHCs as well. Second, through introducing the signaling theory, this study not only reveals the influence mechanism of physicians' knowledge-sharing on patients' engagement but also contributes insights on applying the signaling theory in OHCs context. Third, by investigating the moderating role of registration duration, this study identifies the boundary of the impact of physicians' knowledge-sharing on patients' engagement. This study also provides practical guidance for physicians and OHCs platform managers on how to effectively attract patients through publishing educational articles.

## Literature review

### Physicians' knowledge-sharing in OHCs

Knowledge-sharing in OHCs is defined as the knowledge exchange between physicians and patients in online health communities (16). Knowledge-sharing has gradually become the driving force for the sustainable development of virtual communities, and even the driving force for their success or failure (2). There are two types of physicians' knowledge-sharing, namely paid sharing (private-sharing) and free sharing (public-sharing) (17). The former refers to the private interaction between physicians and patients to meet the health information needs of patients through paid diagnosis and treatment consultation. The latter means that physicians provide free health and medical information by writing popular scientific articles and replies in the community (2, 17).

Scholars have used multiple perspectives to investigate the antecedents of physicians' knowledge-sharing in OHCs (9). Both Lin et al. (18) and Imlawi and Gregg (19) found reputation, shared vision, altruism, self-efficacy positively influenced knowledge-sharing of medical professionals. Similarly, Zhang et al. (13) found that knowledge-sharing willingness of medical professionals was positively correlated with intrinsic and extrinsic motivations. In addition, some scholars have studied the beneficial impact of knowledge-sharing in online health communities. For instance, Bryant et al. (16) found that knowledge-sharing in OHCs can improve physician-patient relationships and health service quality. Similarly, Chen et al. (20) indicated that physicians can benefit from patients and build long-term positive relationships with them when they share knowledge in OHCs.

The literature has some limitations in physicians' knowledge-sharing in OHCs. First, how knowledge-sharing by physicians in OHCs affects patient engagement has received limited research attention. Second, scholars have largely ignored the comparative effects of paid knowledge-sharing and free knowledge-sharing. To address the above research gaps, this study explores the effects of both paid knowledge-sharing and free knowledge-sharing on patient engagement and examines the difference between these effects.

### Patient engagement in OHCs

User engagement has always been a focus issue by scholars and an operational challenge for managers (21). Scholars have defined and explained this concept. For instance, Youngdahl et al. (22) proposed that user engagement is the behavior that users seek in order to get their own satisfaction with the service in term of the level of happiness and participation in activities. Wasko and Di Gangi (23) introduced user engagement as the kind of mind that can ensure enhanced participation and bring meaningful personal benefits.

In addition, many scholars have conducted deeper research, i.e., divide user engagement into levels. Muntinga et al. (24) introduced consumers' brand related activities (COBRAs) to divide the hierarchy of user engagement. In this model, users can perform one or more participation behaviors in consumption, contribution and create. Lagun and Lalmas (25) used taxonomy to divide user engagement into four levels, i.e., bounce, shallow, deep, and complete. Aroused by Lagun's taxonomy and COBRAs framework, Alwash et al. (6) propose two levels of user engagement with brand value propositions—shallow and deep user engagement.

User engagement can effectively improve the development and growth of an OHC platform (26). Therefore, many scholars began to study user engagement in OHCs. Previous studies mainly studied user engagement from two aspects, namely, the physicians' and patients' engagement (21). Although OHC is a physician driven platform (27), many scholars have begun to explore patient engagement in OHCs. For instance, Bansal et al. (28) found that psychological factors are important drivers of patient engagement in OHCs. Besides, Li et al. (29) showed that the status, reputation, and self-representation of physicians promote patient engagement in OHCs. In addition, there may be interaction between physician engagement and patient engagement. There is evidence that patients' participation also stimulates physicians' participation (21, 30).

Madupu and Cooley (31) distinguished active participation and inactive participation in online communities. Inactive participation was simply browsing or reading information in online communities, while active participation involved posting new messages or replying to others' messages. Similarly, some scholars have distinguished patient visit and patient consultation in OHC (32, 33). Patients' visit to physician's homepage was the beginning of getting to know the physician (34), which is a shallow engagement; while patients engage at a deeper level when they comment and consult (35). Many scholars have put patient visit and patient consultation into their researches. Meng et al. (9) took patient visit as the reputation of physicians and explored its impact on physicians' special knowledge sharing. Shah et al. (36) explored the impact of online and offline signals and disease risk on patient consultation. Although scholars have distinguished between patient visit and patient consultation and conducted some studies based on them, only a few scholars have made comparative analysis on them. Therefore, this study introduced patient visit and patient consultation as variables to measure patient engagement, and then examine how educational knowledge-sharing by physicians can vary patient engagement at different levels.

### Signaling theory

Signaling theory can be used to explain the behavior of enterprises or organizations sending relevant signals to reduce the information asymmetry between them and stakeholders, and then obtain the support of stakeholders to improve their benefits (37). The theory is composed of three primary elements, i.e., signaler, receiver, and signal (11). In this theory, the signal is private and can be separated into positive and negative signal information (38). In addition, the signaler possesses more information that the receiver cannot get, and they can decide whether, when, and how to transmit information to the receiver (39, 40). The receiver must take measures to understand the information and distinguish whether the information is efficacious. The efficacious signal usually has two characteristics: observability and cost (40, 41).

Signaling theory has been applied in different research contexts such as e-commerce and hotel management. For instance, Choi et al. (42) introduced reputation, newness, retro features as the internal signal, and proposed review valence, product popularity, price, user engagement as the external signal to explore the impact of these signals on digital video games. Filieri et al. (43) analyzed the moderating role of product quality signals in the relationship between extremely negative ratings and review helpfulness.

Online health communities are the area of significant information asymmetry. In this area, physicians publish free or paid consultations in the platform. Physicians possessing professional medical knowledge have a deeper understanding of the quality of services they provide, so they belong to the information advantage group (44). Patients lacking professional knowledge are unable to assess the quality of the service that they have obtained, so they belong to the information vulnerable group (45).

Due to the high information asymmetry of online medical platform, scholars gradually introduce signal theory into this field. Zhang et al. (4) took online free services and reputation as signals, and found that both can improve physicians' private benefits. Liu et al. (46) found that there was a significant positive correlation between personal reputation, organizational reputation (i.e., signals of physicians), and the amount of physicians' inquiries. Li et al. (47) found that offline status, online reputation, and self-presentation act as signals showing a positive impact on the number of physicians' orders. Educational knowledge-sharing of a physician reflects his/her service competence and benevolence and can be regarded as signals helping patients make choice decisions.

Based on the above review, this study takes a leading OHC in China as the research object, introduces physicians' paid knowledge-sharing and free knowledge-sharing as two signals of physicians' self-representation, then compares the impact of the two signals on patient engagement in OHCs.

## Research model and hypotheses development

**Figure 1:**
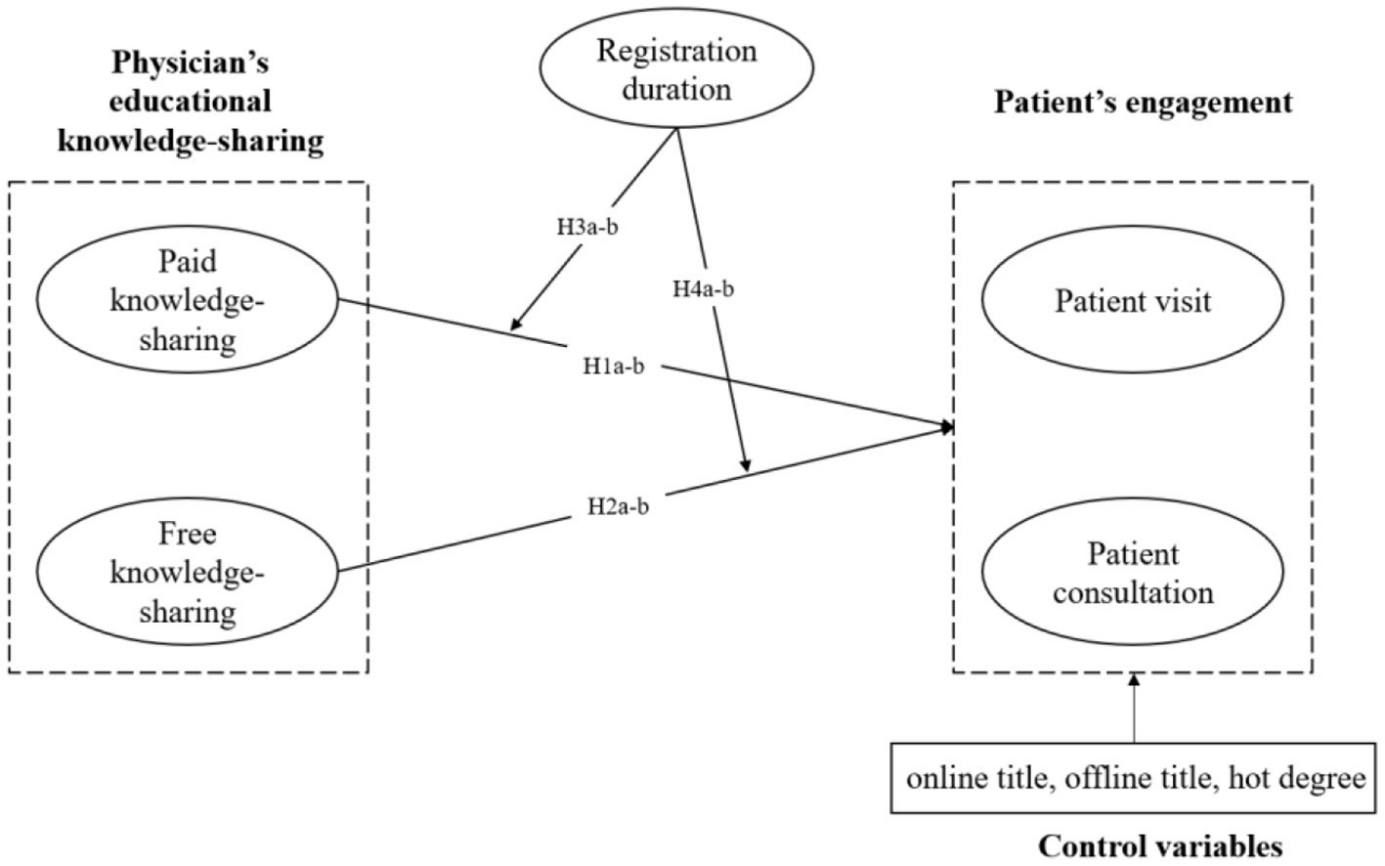
Research model.

The objective of this study is to identify the effects of paid and free educational knowledge-sharing of physicians on patient engagement in OHCs. Particularly, we specified patient engagement as patient visit and patient consultation. The role of physician's registration duration in the relationship between knowledge-sharing and patient engagement was also examined. The research model is proposed as Figure 1.

### Direct effects of knowledge-sharing on patient engagement

Knowledge is a kind of characteristic service (48). Especially, the educational knowledge, can be regarded as a service transferred from individuals with rich professional knowledge to individuals lacking related knowledge. Sharing educational knowledge can fill information asymmetry between offers and receivers. In OHCs, physicians publish some educational articles to spread and share medical knowledge with patients. Research indicated that such knowledge-sharing is important for patient engagement (13). On the one hand, it not only empowers patients with more medical knowledge but enables them to reduce redundant learning efforts (49). On the other hand, through sharing knowledge to patients, physicians can demonstrate their expertise as well as attracting more patients. According to the signaling theory, signalers (i.e., physicians) provide signals (i.e., educated articles) to receivers (i.e., patients) to share their expertise and services, while patients can utilize these signals to make suitable decisions (11, 50).

In this study, paid knowledge-sharing refers to physicians sharing the educational articles that require payment. Patients who want to read the educated articles and learn the medical knowledge need to pay according to the price. Through reading the paid articles, patients can receive high quality medical information, suggestions, or guidance from physicians, which has been found to significantly influence patients' behavior in OHCs (9). From the perspective of signaling theory, physicians' paid knowledge-sharing is a signal to present their expertise to patients (51). Since there is great information symmetry in professional medical knowledge between physicians and patients, it is difficult for patients to evaluate the expertise and professional competence of physicians before engaging in OHCs service. However, the paid knowledge-sharing of physicians is a critical indicator to measure physicians' ability due to the high-quality knowledge. In this regard, patients are likely to form their evaluation on physicians based on the shared knowledge. Specifically, patients are likely to believe physicians who share paid knowledge have strong professional ability. Previous research indicated that professional ability of knowledge contributors has significant influence on user engagement (52). For instance, the more patients trust a physician's ability, the more willing they are to engage in the physician's medical services (53). Specific to this research context, paid knowledge-sharing facilitates patients to trust physicians' professional ability, inducing more patients to visit OHCs services (i.e., patient visit) and purchase OHCs services (i.e., patient consultation). Thus, we propose that:

H1a: Paid knowledge-sharing is positively related to patient visit.
H1b: Paid knowledge-sharing is positively related to patient consultation.

Free knowledge-sharing refers to the sharing of free educational articles by physicians in OHCs. Anyone (including all patients and physicians) can browse and read these articles without payment. Physicians' free knowledge-sharing is a voluntary help and service offered to patients, aside from an egocentric profit motive (54). In previous studies, such free service is regarded as a signal of physicians' positive participation in OHCs, which will influence how patients think about physicians' services (4). Similarly, free knowledge-sharing acts as a signal demonstrating how much physicians are willing to participate in OHCs and offer help. Apparently, in the process of sharing free knowledge, physicians do attract more patients to engage in OHCs. On one hand, physicians' positive participation in OHCs means that they are likely to offer prompt and reliable services, which may draw more traffic to their home pages. On the other hand, sharing free knowledge shows a physician' kindness and benevolence, which may enhance patients' trust in him/her, and thus it is more likely that patients will be willing to select this physician for consultation (55). Thus, it is reasonable that physicians' free knowledge-sharing on OHCs will facilitate patients to visit their home pages and purchase their consultation services. Based on this, we propose that:

H2a: Free knowledge-sharing is positively related to patient visit.
H2b: Free knowledge-sharing is positively related to patient consultation.

### Moderating effects of physicians' registered duration

Information asymmetry between physicians and patients has been stressed as a critical issue in OHCs due to the medical profession (4, 46). In this context, the paid or free knowledge shared (i.e., educational articles) by physicians send a signal to patients that the physician has the potential to provide satisfactory services. However, the signal may not be the only factor influencing patients when they make decisions about visiting or consulting a physician. Since the signal is sent from signaler (i.e., physicians) to receivers (i.e., patients), characteristics of signalers may affect how receivers process the signal.

The registration duration of a physician refers to how early and long physicians offer service at OHCs. Early service time shows that physicians are innovative in providing multichannel services and long service time indicates that physicians are more qualified to provide online services. Thus, registration duration can be regarded as an indicator reflecting the physician's online service innovation and qualification. Since signalers' identity and characteristics denote how credible and reliable of their signals (11, 51), registration duration of physicians may influence patients' understanding of physicians' signals. Thus, registration duration of physicians may play an important role in the relationship between physicians' knowledge-sharing and patient engagement. For example, for a physician registered at OHCs for a long time, patients are prone to believe that his/her signal is more credible. In this case, compared with physicians registered at OHCs in later time, the knowledge (i.e., the signal) shared by physicians registered at OHCs earlier is likely to bring stronger impacts on patient engagement.

In this study, paid knowledge-sharing of a physician is a signal reflecting his/her service quality and competence (56), and physician's registration duration may influence how patients interpret the signal. The length of duration for a physician registered at OHCs indicates the online service qualification level of this physician, which will enhance patients' trust on the physician and attract more visits by patients. When a physician shared paid knowledge (i.e., sending a signal) on OHCs, a long registration duration may strengthen the impact of this signal. That is to say, patients are more likely to believe that the paid knowledge shared by a physician registered at OHCs for a long time is more trustworthy and useful, and thus, they will be motivated to visit the physician's home page. Moreover, based on the high quality sharing of paid knowledge, the long registration duration of a physician can entrust patients with greater confidence that the physician has adequate competence and qualification to provide satisfactory services (54). In this case, when patients decide to consult a physician after reading the shared paid knowledge, they are prone to select a physician registered at OHCs for a long time. Thus, the longer a physician' registration duration, the greater the impacts of paid knowledge-sharing on patient engagement. Thus, we propose that:

H3a: Physician's registration duration can strengthen the effect of paid knowledge-sharing on patient visit.
H3b: Physician's registration duration can strengthen the effect of paid knowledge-sharing on patient consultation.

Similarly, physicians' registration duration may also moderate the effects of free knowledge-sharing on patient engagement. Free knowledge-sharing of a physician is a signal indicating his/her willingness to offer help and benevolence in providing medical services (57). Although the free knowledge-sharing can attract patients to visit the physician's home page, a long registration duration makes physician's benevolence more credible and thus bring more traffic on service visit. In addition, for a physician who newly registers at OHCs, sharing free knowledge may be an operating strategy to attract patients. Thus, it is lacking the clue that this physician will behave friendly during the consultation service. However, for a physician who has registered at OHCs for a long time, he/she is still active in sharing free knowledge is largely a sign of kindness. As such, patients are prone to trust that this physician can provide satisfactory services and prefer him/her for consultation (55). Thus, compared with physicians newly registered at OHCs, free knowledge-sharing of earlier physicians has stronger impacts on patient engagement. Based on this, we propose that:

H4a: Physician's registration duration can strengthen the effect of free knowledge-sharing on patient visit.
H4b: Physician's registration duration can strengthen the effect of free knowledge-sharing on patient consultation.

## Research methodology

### Data collection

This research was based on data collected from a leading online health community in China. As of December 2021, nearly 3,00,000 physicians have entered the community, and more than 70 million patients have also registered on the community. The main reasons for choosing this platform as the research object are explained as follows: (1) the community has a large number of physicians and patients, which can collect rich data; (2) physicians in this community can choose to publish paid articles and free articles, which is consistent with our research context; (3) patients in this community can choose to visit physicians' home pages or consult a physician for medical services. Thus, the OHC is a suitable context to examine our research model and hypotheses.

On this community, physicians provide links to their home pages, through which patients can learn about physicians' basic information (e.g., name, hospital, title, and registration duration). Patients can choose to consult physicians for personalized diagnose and treatment. The number of patients who visited and consulted a physician will be displayed on the home page of the physician. At the same time, physicians also publish some educational articles about medical knowledge in community. There are some paid articles that requires a fee to read, and some free articles provided for the public freely.

We collected data of article publishing and home pages information of physicians in the community using a Java-based program. Finally, data of 1,68,377 physicians were obtained. For the research model, we used the number of shared paid articles as the measure of physicians' paid knowledge-sharing behavior and the number of shared free articles as the measure of physicians' free knowledge-sharing behavior. Moreover, patient visit was reflected by the number of patients who have visited the physician's home page, and patient consultation was measured by the number of patients who have consulted the physician. Registration duration was defined as the length of time a physician has been affiliated with the OHCs, which was measured by the data capture time minus the time the physician opened an account. Overview of variables was in Table 1.

**Table 1:** Overview of variables.

**Variables**		**Description**	**Mean**	**SD**	**Min**	**Max**
Dependent variables	Patient visit	No. 10 of thousands of patient visits of physician	112.558	368.7878	0.0016	14948.56
	Patient consultation	No. 10 of thousands of patient consultations of physician	0.1523	0.3056	0.0001	5.9934
Independent variables	Particles	No. paid articles of physician	0.414	14.28	0	4,716
	Farticles	No. free articles of physician	2.816	8.991	0	1,748
Moderator variables	Time	Registration duration of physician's account	96.129	46.263	3.933	162.233
Control variables	doc_title	No. titles of physician	0.0316	0.306	0	8
	doc_hot	Hot degree of physician	2.439	1.400	0	8
	doc_prof	No. offline titles of physician	2.550	1.186	1	4

In addition, we tested the discriminant validity of the measures. The correlation matrix of the measures was in Table 2. The results show that most of the correlations between any two variables were <0.700 (except the correlation between patient consultation and patient visit). These results indicate the discriminant validity of the measures.

**Table 2:** Correlation matrix of the measures.

**Variables**	**(1)**	**(2)**	**(3)**	**(4)**	**(5)**	**(6)**	**(7)**	**(8)**
(1) Patient visit	1.000							
(2) Patient consultation	0.810	1.000						
(3) Farticles	0.015	0.019	1.000					
(4) Particles	0.012	0.044	0.111	1.000				
(5) Time	0.298	0.228	−0.018	−0.005	1.000			
(6) doc_title	0.377	0.536	0.005	0.008	0.095	1.000		
(7) doc_hot	0.314	0.463	0.008	0.002	0.245	0.135	1.000	
(8) doc_prof	0.171	0.171	0.007	−0.005	0.497	0.065	−0.104	1.000

### Data analysis and results

To test the hypothesis that different types of physicians' knowledge-sharing will affect patients' engagement, two empirical models were developed as follows:


(1) $$\begin{array}{l} patient\;visit\;=\;\beta 0+\beta 1Particles+\beta 2Farticles\;\\ \;+\beta 3Particles^{*}time+\beta 4Farticles^{*}time\\ \;+\beta^{\prime }Z \end{array}$$

(2) $$\begin{array}{l} patient\;consultation\;=\;\beta 0+\beta 1Particles+\beta 2Farticles\;\\ \;+\beta 3Particles^{*}time+\beta 4Farticles^{*}time\\ \;+\beta^{\prime }Z \end{array}$$

where β is the coefficient, and Z is the variable that controls Particles and Farticles, include doc_title, doc_hot and doc_prof. Model (1) tests the effect of paid and free knowledge-sharing on patient visit as well as the effect of physician's registration duration on the relationship. Model (2) tests the effect of paid and free knowledge-sharing on patient consultation as well as the effect of physician's registration duration on the relationship.

In this paper, the fixed effect model is used to test the model. In the first stage, Model (1) and Model (2), only with control variables, were tested for comparison purposes. In the second stage, Model (1) and Model (2), without moderator variables, were tested to explore the impact of free knowledge-sharing and paid knowledge-sharing on patient visit and patient consultation. In the third stage, Model (1) and Model (2), with interactive items, were tested to verify its moderating effect. The results are given in Table 3.

**Table 3:** Hierarchical regression results.

	**Stage1**		**Stage2**		**Stage3**	
	**Patient visit**	**Patient consultation**	**Patient visit**	**Patient consultation**	**Patient visit**	**Patient consultation**
Particles			0.2063^***^ (0.0572)	0.0011^***^ (0.0001)	−0.6443^*^ (0.3455)	0.0017^***^ (0.0003)
Facticles			0.4440^***^ (0.0909)	0.0003^***^ (0.0001)	−8.079^***^ (0.2563)	−0.0039^***^ (0.0002)
doc_title	0.2626^***^ (0.0020)	0.0002^***^ (0.0000)	0.2625^***^ (0.0020)	0.0002^***^ (0.0000)	0.2613^***^ (0.0023)	0.0002^***^ (0.0000)
doc_hot	0.1333^***^ (0.0020)	0.0002^***^ (0.0000)	0.1333^***^ (0.0020)	0.0002^***^ (0.0000)	0.1140^***^ (0.0024)	0.0002^***^ (0.0000)
doc_prof	0.3175^***^ (0.0094)	0.0001^***^ (0.0000)	0.3174^***^ (0.0094)	0.0002^***^ (0.0000)	0.3716^***^ (0.0122)	0.0001^***^ (0.0000)
ParticlesXtime					0.0148^***^ (0.0034)	−5.54e-06^**^ (0.0000)
FarticlesXtime					0.1348^***^ (0.0034)	0.0001^***^ (0.0000)
Constant	−431.77^***^ (5.9633)	−0.5212^***^ (0.0054)	−432.9^***^ (5.9664)	−0.5222^***^ (0.0054)	−385.4^***^ (7.5833)	−0.5140^***^ (0.0054)
*N*	1,68,372	1,15,231	1,68,372	1,15,231	1,33,822	1,15,230
*R*-squared	0.1836	0.3677	0.1838	0.3689	0.1836	0.3727

* p < 0.05,

** p < 0.01,

*** p < 0.001.

In Stage 1, we only introduced control variables as control group to facilitate the exploration of direct and moderating effects. In Stage 2, we found that Particles (β = 0.2063, *t* = 3.61, *p* < 0.001) and Farticles (β = 0.4440, *t* = 4.89, *p* < 0.001) positively and significantly influence patient visit. Therefore, H1a and H2a (both free knowledge-sharing and paid knowledge-sharing are positively related to patient visit). In addition, we found that Particles (β = 0.0011, *t* = 14.1, *p* < 0.001) and Farticles (β = 0.0003, *t* = 3.62, *p* < 0.001) positively and significantly influence patient consultation. Therefore, H1b and H2b (both free knowledge-sharing and paid knowledge-sharing are positively associated with patient consultation).

**Figure 2:**
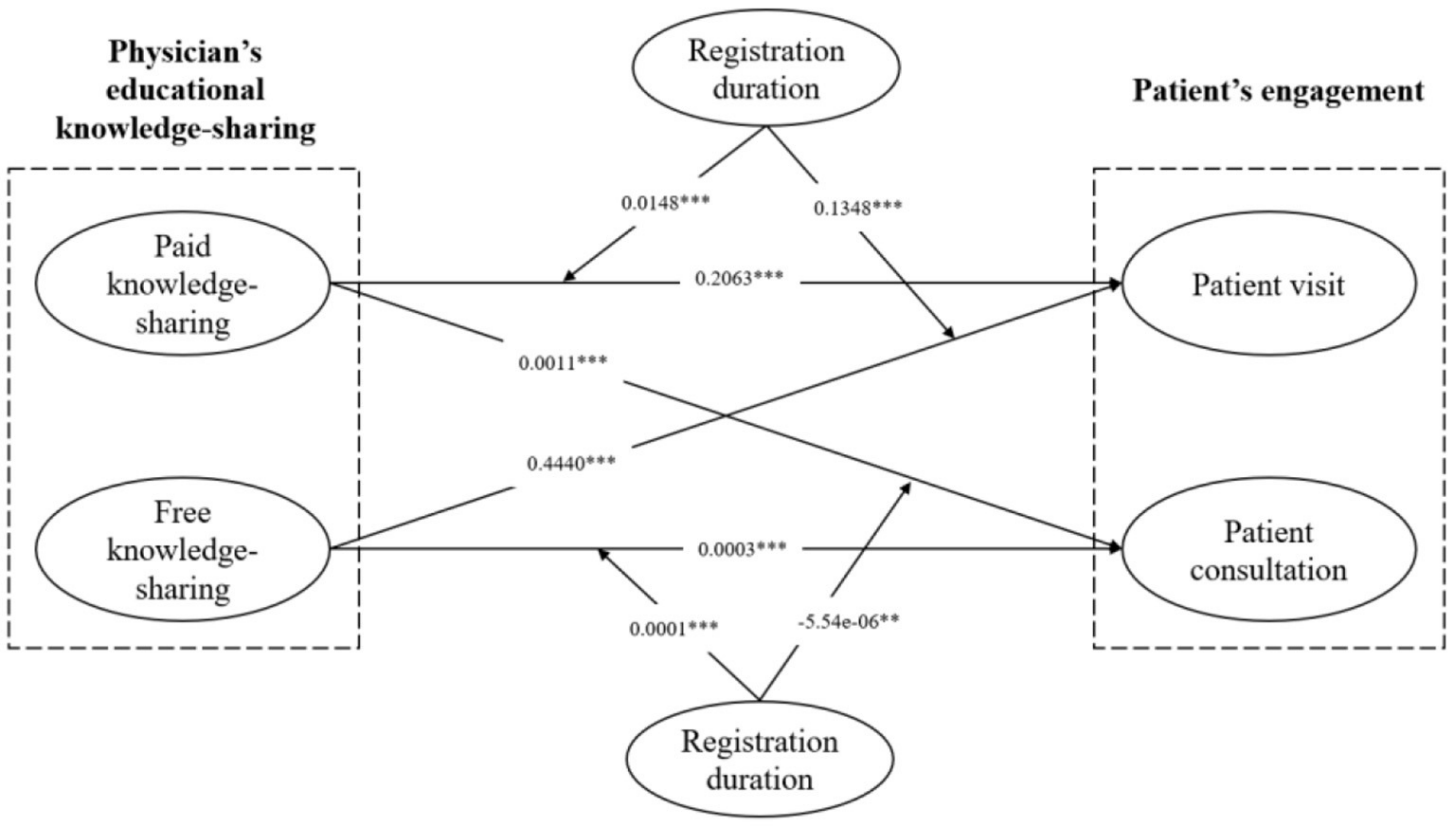
Results diagram.

In Stage 3, we tested the moderator effect of physician's registration duration. We found that, for patient visit, the interaction term ParticlesXtime (β = 0.0148, *t* = 4.37, *p* < 0.001) and FarticlesXtime (β = 0.1348, *t* = 39.07, *p* < 0.001) were positive and significant; while for patient consultation, the interaction term ParticlesXtime (β = −5.54e-06, *t* = −2.07, *p* < 0.01) was negative and significant and FarticlesXtime (β = 0.0001, *t* = 25.99, *p* < 0.001) was positive and significant. Therefore, H3a, H4a, and H4b (physician's registration duration can strengthen the impact of paid knowledge-sharing on patient visit; physician's registration duration can strengthen the impacts of free knowledge sharing on both patient visit and patient consultation) were supported. H3b (physician's registration duration can strengthen the impact of paid knowledge sharing on patient consultation) was rejected. Figure 2 presents the relationships between variables and Table 4 shows how all the hypotheses are supported.

**Table 4:** Summary of hypotheses support.

**Hypotheses**	**Influence route**	***P*** **-value**	**Hypotheses support**
H1a	Paid knowledge-sharing → patient visit	^***^	Supported
H1b	Paid knowledge-sharing → patient consultation	^***^	Supported
H2a	Free knowledge-sharing → patient visit	^***^	Supported
H2b	Free knowledge-sharing → patient consultation	^***^	Supported
H3a	Paid knowledge-sharing^*^registration duration → patient visit	^***^	Supported
H3b	Paid knowledge-sharing^*^registration duration → patient consultation	^**^	Not supported
H4a	Free knowledge-sharing^*^registration duration → patient visit	^***^	Supported
H4b	Free knowledge-sharing^*^registration duration → patient consultation	^***^	Supported

* p < 0.05,

** p < 0.01,

*** p < 0.001.

### Additional analysis

To further clarify the effects of the two types of knowledge-sharing and verify whether there is difference between these two kinds of effects statistically, we conducted additional analyzes to compare the impacts of free knowledge-sharing and paid knowledge-sharing on patient visit and patient consultation, respectively. According to Flannery and Rangan (58), we calculated the dominance impact of free knowledge-sharing and paid knowledge-sharing. The results are presented in Table 5. The results show that for patient visit, free knowledge-sharing has a greater positive impact than paid knowledge sharing; while for patient consultation, paid knowledge sharing has a greater positive impact than free knowledge sharing.

**Table 5:** Dominance analysis results.

	**Patient visit**	**Patient consultation**
Particles	0.000118	0.00180
Farticles	0.000219	0.000267
*N*	1,68,377	1,15,236

## Discussion

### Key findings

There are several key findings in this study. First, the results indicated that physicians' educational knowledge-sharing has significant direct impacts on patients' engagement in OHCs. Particularly, both paid knowledge-sharing and free knowledge-sharing not only encourage patients to visit physician's home page but also lead them to purchase consultation service. These results are consistent with the mechanism of the signaling theory, which indicates that signal receivers (i.e., patients) utilize signals (i.e., knowledge-sharing) to help them make decisions (i.e., engagement in OHCs) (51).

Second, through comparing the effects of paid knowledge-sharing and free knowledge-sharing on patient engagement, we found that two types of knowledge-sharing show relative importance in inducing patient visit and patient consultation. This study complements prior research confined to one type of knowledge-sharing (54, 59). Specifically, paid knowledge-sharing shows stronger impact than free knowledge-sharing on patient consultation, while free knowledge-sharing plays more important roles than paid knowledge-sharing in facilitating patient visit. This is reasonable since paid knowledge-sharing signals physicians' service competence, while free knowledge-sharing signals physicians' kindness and benevolence. When patients need to consult a physician, they pay more attention to the physician's service ability and competence. In contrast, when patients only visit home pages to learn physicians' information, they prefer kind and friendly physicians.

Third, the results verified the moderating role of physician's registration duration to engage patients in OHCs. The findings show that for physicians who has registered at OHCs for a longer time, their free knowledge-sharing has stronger impacts on patient visit and patient consultation. However, for paid knowledge-sharing, registration duration shows different moderating effects. Specifically, registration duration strengthens the impact of paid knowledge-sharing on patient visit but weakens the impact of paid-knowledge-sharing on patient consultation. This means that for physicians with high level of service innovation and qualification, through publishing paid article, they perform better in attracting patients visit but perform worse in attracting patients consultation. Also, the results are in line with the signaling theory, which asserts that signalers' characteristics influence receivers' understanding of signals (11, 51).

### Theoretical implications

This study contributes knowledge to the literature in several ways. First, this study extends current research on patient engagement in OHCs. Different from prior research which failed to conceptualize specific patient engagement behavior in OHCs (7), this study identifies physicians' shallow (i.e., patient visit) and deep (i.e., patient consultation) engagement behavior. Furthermore, to the best of our knowledge, this study is the first to explore the effects of physicians' educational knowledge-sharing on patient engagement in OHCs. Although factors of patient engagement in OHCs have been studied, they only focused on patients' perceptions lacking attention on the role of physicians' behavior (7, 60). This leaves a research gap to consider how physicians' behavior influence patient engagement since physicians and patients are the two main participants of OHCs. Through addressing the educational knowledge-sharing of physicians in OHCs, this study not only fills up the research gap but also provides a new direction for future research on patient engagement in OHCs.

Second, through drawing upon the signaling theory, this study reveals how physicians' educational knowledge-sharing influence patients' engagement and contributes to the signaling theory in the OHCs context as well. We found that paid knowledge-sharing has stronger effect on patient deep engagement while free knowledge-sharing has stronger effect on patient shallow engagement. It is one of the first studies that empirically compared the impacts of different types of knowledge-sharing on patient engagement, echoing (11) appeal that further research should pay more attention on how to signal to reach an optimal effect. Accordingly, introducing the signaling theory, this study provides a deeper understanding of the effects of physicians' educational knowledge-sharing. Moreover, it also enriches the signaling theory in OHCs context by demonstrating the effects of signals generated from physicians' educational knowledge-sharing behavior.

Third, this study identifies the critical role of physicians' registration duration in the relationship between physicians' educational knowledge-sharing and patient engagement in OHCs. Physicians' characteristics, such as seniority and ranking, have been well-testified to play important role in previous OHCs research (4, 14). However, physicians' registration duration, one of physician's characteristics representing the online service innovation and qualification of a physician, has rarely been examined. Taking physicians' registration duration as a moderator, we found that the educational knowledge-sharing of physicians who has entered OHCs for a longer time shows stronger impacts on patient engagement. That is, a long registration duration will strengthen impacts of a physician's educational knowledge-sharing. The results clarify the boundaries of the effects of knowledge-sharing as a signal on patient engagement. In this regard, this study provides an insight into the signaling theory by considering boundaries and guides future research to keep their eyes on the effect of physicians' registration duration.

### Practical implications

This study provides some insights for practitioners, especially for physicians and OHCs platform managers. First, this study suggests physicians and platform managers to pay more attention to patient engagement in OHCs, which is the foundation of platform development. Patient engagement is specified into two different types: patient visit and patient consultation. Thus, physicians and platform managers are supposed to notice the difference and take corresponding measures to promote each kind of engagement.

Second, this study has verified that educational knowledge-sharing of physicians has significant positive impacts on patient engagement in OHCs, including patient visit and patient consultation. Thus, to attract more patients, physicians are suggested to share knowledge actively. For example, the platform can develop incentive to encourage physicians publishing more educational articles. Moreover, since paid knowledge-sharing and free knowledge-sharing show relative impacts on patient visit and patient consultation, physicians can adjust their knowledge-sharing behavior for different purposes. For instance, when they aim to attract more patients to purchase consultation services, they should pay more attention to publishing paid educational articles.

Third, the results indicated that the length of physicians' registration duration can strengthen the impacts of knowledge-sharing on patient engagement. That is to say, physicians who have long-lasting registration at OHCs perform better than those newly registered physicians in attracting patients with sharing educational articles. Particularly, platform managers and physicians should notice that for sharing paid educational articles, long time-registered physicians and newly registered physicians perform equally in attracting patient consultation but differently in attracting patient visit. The results can guide platform managers and physicians to formulate strategies in publishing educational articles according to physicians' registration time.

### Limitations and directions for future research

There are several limitations for interpreting the results in this study. First, the data were collected from only one OHC in China. Although the community is one of the largest OHCs in China, the generation of research findings is limited for other health systems and cultural contexts. Therefore, future research is suggested to collect data from different platforms in various cultural contexts for replication. Second, the research model and hypotheses were examined with cross-sectional data. To increase the robustness of research findings, future research can use panel data to test the hypotheses with a dynamic perspective. Third, this study only examined the moderating effect of physicians' registration duration. Whether physicians' other characteristics play a role in the relationship between physician knowledge-sharing and patient engagement is another direction to extend this line of research.

## Conclusion

This study empirically examined the effects of physicians' educational knowledge-sharing on patient engagement in OHCs and identified the moderating role of physicians' registration duration. The results verified that both paid and free knowledge-sharing have positive effects on patients' shallow engagement (i.e., visiting physicians' home pages) and deep engagement (i.e., consulting medical service). Particularly, we found that paid knowledge-sharing shows stronger impact on deep engagement while free knowledge-sharing has greater effect on shallow engagement. This finding extends our understanding of the relationship between physicians' educational knowledge-sharing and patients' engagement. The results regarding the moderating effect of physicians' registration duration indicate that knowledge-sharing of physicians who have registered at OHCs for a longer time plays more important roles in inducing patient engagement. These results not only notably contribute to literature on engagement and signaling theory but also provide practical suggestions for physicians and OHCs platform managers on attracting patient engagement in OHCs.

## Data availability statement

The original contributions presented in the study are included in the article/supplementary materials, further inquiries can be directed to the corresponding author/s.

## Author contributions

XM and PZ: conceptualization, methodology, and writing. FM: conceptualization, writing, and editing. K-hL: review, editing, and supervision. All authors contributed to the article and approved the submitted version.

## Funding

This study was funded by the National Natural Science of China (72001094).

## Conflict of interest

The authors declare that the research was conducted in the absence of any commercial or financial relationships that could be construed as a potential conflict of interest.

## Publisher's note

All claims expressed in this article are solely those of the authors and do not necessarily represent those of their affiliated organizations, or those of the publisher, the editors and the reviewers. Any product that may be evaluated in this article, or claim that may be made by its manufacturer, is not guaranteed or endorsed by the publisher.
